# Renal Cell Carcinoma in Native Kidney After Kidney Transplantation: A Multicenter Case Control Study With a Focus on Screening Strategy

**DOI:** 10.3389/ti.2025.14487

**Published:** 2025-06-16

**Authors:** Pierre Pommerolle, Maryam Assem, Marine Uhl, Philippe De Sousa, Dominique Guerrot, Marc Hazzan, Thierry Lobbedez, Ophélie Fourdinier, Gabriel Choukroun

**Affiliations:** ^1^ Department of Nephrology, Dialysis, and Transplantation, Amiens University Hospital, Amiens, France; ^2^ MP3CV Research Unit, University of Picardie Jules Verne, Amiens, France; ^3^ Department of Nephrology, Tanger University Hospital, Tanger, Morocco; ^4^ Department of Urology and Transplantation, Amiens University Hospital, Amiens, France; ^5^ Department of Nephrology, Dialysis, and Transplantation, Rouen University Hospital, Rouen, France; ^6^ Department of Nephrology, Dialysis, and Transplantation, Lille University Hospital, Lille, France; ^7^ Department of Nephrology, Dialysis, and Transplantation, Caen University Hospital, Caen, France

**Keywords:** renal cell carcinoma, native kidney, kidney transplantation, screening, survival

## Abstract

Renal cell carcinoma (RCC) of native kidney is more prevalent after kidney transplantation than in the general population. Risk factors and the value of screening remain unclear. We conducted a multicenter case-control study in kidney transplant recipients transplanted between 1989 and 2017. All patients with RCC were included, and two controls were matched to each case. Two centers performed annual screening (AnS group) and the other two had other strategies (OS group). A total of 125 cancers were found in 113 patients. The majority of cancers were stage T1-T2 (92.0%), 1.6% had metastasis at diagnosis and ten (9.0%) had recurrence after nephrectomy. Men [OR 2.2; IC 95% (1.2–4.4); p = 0.02] and acquired cystic kidney disease [OR 3.2; IC 95% (1.8–5.9); p < 0.01] were associated with cancer in multivariate analysis. The 10-year survival was poorer in cases (65.6% vs. 79.1%, p < 0.001). The AnS group had fewer relapses (5.0% vs. 18.2%, p = 0.02) and a lower rate of cancer-related deaths (16.0% vs. 46.1%, p = 0.04). Survival of patients with RCC is lower than in control patients. Annual screening could improve cancer prognosis, its benefit needs to be evaluated in larger studies.

## Graphical Abstract

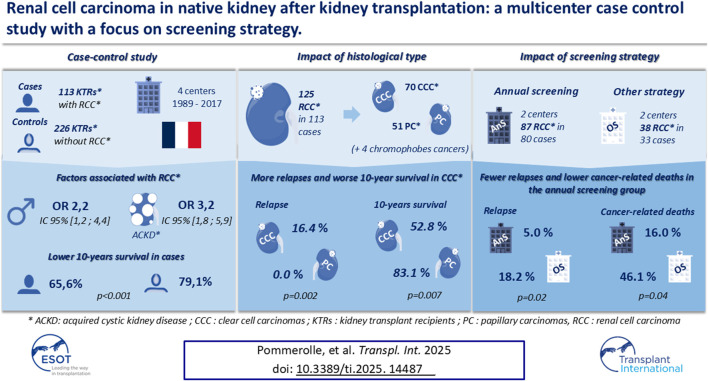

## Introduction

Kidney transplantation (KT) is the treatment of choice for chronic kidney disease (CKD), offering, in comparison to dialysis, a better quality of life and survival. However, the use of immunosuppressive treatments increases the risk of post-transplantation complications, mostly infections and cancers. Thus, the risk of cancer is 2 to 5-fold higher in kidney transplant recipients (KTRs) than in the general population [1, 2], and 20% of KTRs will develop cancer within 10 years after KT [3]. A higher risk is observed, compared to the general population, for skin cancer, Kaposi’s sarcoma, post-transplantation lymphomas, and urological malignancies particularly the renal cell carcinoma (RCC) of native kidney [1, 2].

Every year, RCC affects 350,000 new patients worldwide and 30,000 patients die from their cancer [4]. The main risk factors for RCC are age older than 60 [5], male gender [5], smoking [6], high blood pressure [7–9], obesity [10, 11], and CKD [12]. Acquired cystic kidney disease (ACKD) is a multi-cystic differentiation of native kidneys, mainly affecting patients with end-stage of CKD. ACKD is suspected to be a risk factor for RCC, due to the strong association between ACKD and CKD-patients with RCC (70%–90%) [13]. Treatment for RCC is usually surgical but medical treatment (in particular the use of immunotherapies and tyrosine kinase inhibitors) has clearly improved prognosis of patients with metastatic disease [14, 15].

Several studies, generally retrospective, monocentric, and with small numbers of patients, have studied the risk of RCC in KTRs [16–27]. RCC is 15-fold higher in KT than in the general population [28, 29], and occurred in 0.7% of KTRs [30]. Interestingly, several studies have suggested that the frequency of RCC may be higher when systematic screening is performed (2.7%–4.6%) [18, 21, 23]. The explanations for this higher frequency in KT have not been optimally studied, due to a lack of studies comparing the characteristics of KTRs with RCC to those of KTRs without cancer. The impact of immunosuppressive regimen notably, remains unclear [31]. Finally, there is no consensus on systematic screening of RCC in KTRs. Indeed, the American Society of Transplantation and the Kidney Disease Improving Global Outcomes do not recommend systematic screening [2, 32], while the European Association of Urology recommends performing an annual ultrasound of the native kidneys [33]. The aim of this study is to determine the characteristics of KTRs with RCC in a large population of transplant patients. To evaluate the risk factors of RCC in KT, we compared characteristics of this population to a healthy and matched population of KTRs. Finally, we evaluated the outcome of KTRs with RCC according to the screening strategy of their center.

## Patients and Methods

### Study Design

We conducted a multicentric, retrospective, case-control study in four University Hospitals in France (Amiens, Caen, Lille and Rouen). The study included KTRs transplanted between April 1989 and December 2017. The end of the follow-up was 31 January 2019.

### Selection of Cases and Controls

All patients with a diagnosis of RCC during graft follow-up were included, while patients with a diagnosis of benign tumor of native kidney, graft cancer, or urinary tract malignancy were not. Cases were selected in all centers by the search for “Cancer of native kidney” or “Kidney carcinoma” in the CRISTAL database (Agence de la Biomedecine, Paris, France) and supplemented by questioning the Pathology Department of each center. Finally, the analysis of medical records ensured the diagnosis of RCC. Controls were selected from KTRs transplanted over the same period. All patients underwent pre-transplant evaluation, including abdominal imaging, to rule out active malignancy and RCC. We assigned two controls per case, matching by center, year of transplantation (±2 years), and age at transplantation (±2 years). Cases had to be alive with a functioning graft at the time of the case’s cancer onset and anephric controls were excluded. These criteria was verified in the patient’s medical records.

### Patients’ Medical Records

All data were collected retrospectively from the patients’ medical records. The variables collected included sex, age at transplantation, age at cancer diagnosis (for cases), the number of transplants received, the type and duration of dialysis treatment before KT, cancer history, the presence of a single native kidney, smoking (current or former), obesity, diabetes mellitus, presence of ACKD, cause of end-stage renal disease (ESRD), estimated glomerular filtration rate (eGFR), immune complications (biopsy-proven graft rejection, rejection type and *de novo* donor-specific antibodies [DSA]), immunosuppression characteristics (regimen, drugs, blood calcineurin inhibitor level, and the dose of mycophenolate mofetil received), the histologic characteristics of cancers, cancer treatments (including initial treatment, treatment of recurrence and changes in immunosuppressive therapy). Single native kidney was defined as a single anatomical kidney (acquired or congenital) before KT; obesity was defined as a body mass index >30 kg/m^2^; eGFR was calculated using the Modification of Diet in Renal Disease equation [34]; ACKD was defined as the presence of at least three single cysts in each native kidney on imaging tests (ultrasound, CT scan or magnetic resonance imaging), patients with autosomal dominant polycystic kidney disease (ADPKD) and those without available imaging examinations were excluded from this analysis. Tumors were classified by histological type according to current classifications the year of the discovery of the cancer, and according to the classifications of Fuhrman or International Society of Urological Pathology (based on date of diagnosis) for the tumor grade [35, 36]. The stage of the disease was evaluated according to the Union for International Cancer Control TNM 2017 classification [37, 38].

### RCC Screening

We defined two groups of patients according to the screening strategy of each center: the “annual screening group” (AnS, including patients from Amiens and Rouen) and the “other strategy group” (OS, including patients from Caen and Lille). The annual screening strategy involved alternating CT scans and ultrasounds in Amiens center (alternately) and only an ultrasound screening in Rouen center. The use of iodinated contrast agent for CT scans was permitted but left to the discretion of the nephrologist. In the OS group, Caen center carried out ultrasound at 1 year of KT then every 3 years, while Lille center did not perform systematic screening. Patients in both groups (Ans and OS) could undergo additional imaging, after screening or incidental diagnosis.

### Statistical Analysis

The normality tests used were the Shapiro-Wilk test and the Kolmogorov Smirnov test. In case of normal distribution, means and standard deviations were used to describe continuous variables. Otherwise, we described the results with the median and the interquartile range (IQR). For unpaired subjects, the means were compared by Student’s t-test or the Mann-Whitney (depending on whether the variables were normally distributed or not), and frequencies by the chi-square test or the Fisher test. For matched subjects, frequencies were compared by the Cochran-Mantel-Haenszel test. Survival data (patient survival and graft survival) were evaluated at 10 years from baseline. Baseline corresponded to the date of cancer diagnosis in the cases, and the date of cancer of the matched case for the controls. For patients who had contralateral RCC, only the date of the first cancer was used in the analyses. Graft survival data were censored at the time of recipient death. Survival was analyzed by the Kaplan Meier method. Survival curves were compared by the Logrank test. The odds ratios, described with a 95% confidence interval, were calculated in a bivariate analysis, then included in a multivariate model if p was less than 0.1 using a logistic regression model. The results were considered significant for a p-value less than 0.05. Statistical analyses were performed using SPSS^®^ software (version 21 SPSS Inc., Chicago, IL, United States) and Graphpad Prism (Graphpad Software San Diego, California, United States).

### Ethics

The study followed the 1964 Declaration of Helsinki and its later amendments. In line with the French legislation on retrospective, non-interventional studies, this study was reviewed and approved by an ethic committee (Comité de Protection des Personnes Nord Ouest II, n°2011/17), all patients were informed about the collection of their data and were free to decline participation in the study. The study was registered with the French National Data Protection Commission (Commission Nationale de l’Informatique et des Libertés, Paris, France; registration number: CNIL MR001: n°1449904).

## Results

### Comparison of Cases and Controls

Seven thousand and eighty-four patients were transplanted in the four centers between April 1989 and December 2017 (1511 in Amiens, 1377 in Caen, 2887 in Lille, 1449 in Rouen). One hundred and thirteen patients had a RCC during the study period, i.e., a prevalence of 1.6% in the entire population. The characteristics of the patients are summarized in Table 1.

**TABLE 1:** Cases and controls characteristics.

Variables	Patients		
	Cases n = 113	Controls n = 226	p
Male	87 (77.0)	143 (63.3)	0.03
Age at transplantation	51.3 (10.9)	51.2 (10.9)	ns
Age at baseline	56.1 (10.6)	56.1 (10.7)	ns
Smoke	39 (33.6)	75 (33.2)	ns
Body mass index, kg/m^2^	26.1 (23.4–28.9)	25.9 (22.8–28.5)	ns
Obesity	23 (20.4)	43 (19.0)	ns
Diabetes mellitus	21 (18.6)	40 (17.7)	ns
Acquired cystic kidney disease^a^	52 (50.0)	39 (24.1)	0.01
Single native kidney	12 (10.6)	23 (10.2)	ns
Dialysis before transplantation Hemodialysis	106 (93.8) 94 (88.7)	211 (93.4) 185 (87.7)	ns ns
Time to dialysis, months	27.2 (16.6–45.6)	23.7 (14.8–38.5)	ns
Time to dialysis >3 years	40 (35.4)	58 (25.7)	ns
eGFR at baseline, mL/min/1.73 m^2^	47.0 (36.5–60.0)	46.0 (35.0–57.0)	ns
Prior cancer history History of pre-transplant RCC	11 (9.7) 2 (1.8)	14 (6.2) 0 (0.0)	ns ns
Prior transplantation	16 (14.2)	20 (8.8)	ns
Cause of ESRD Glomerulonephritis Nephroangiosclerosis Diabetes mellitus ADPKD Chronic Interstitial Disease Urologic malformation Other Unknown	55 (48.7) 14 (12.4) 8 (7.1) 7 (6.2) 7 (6.2) 4 (3.5) 8 (7.1) 10 (8.8)	84 (37.2) 10 (4.4) 10 (4.4) 52 (23.0) 14 (6.2) 25 (11.1) 8 (3.5) 23 (10.2)	0.04 0.02 ns 0.01 ns 0.04 ns ns

Data are expressed as mean (standard deviation) or median (interquartile range) for continuous variables (depending on the normal distribution or not) and number (percentage) for categorial variables. eGFR, estimated glomerular filtrate rate; RCC, renal cell carcinoma; ESRD, end stage renal disease; ADPKD, autosomal dominant polycystic kidney disease; ns, non-significant.

^a^ Patients with autosomal dominant polycystic kidney disease and those for whom imaging examinations were not available were excluded from the analysis of this variable.

Cases and controls mostly received a graft from a deceased donor (98.2% in cases and 97.8% in controls). Cases were more likely to be men (77.0% vs. 63.3%; p = 0.03) and to have an ACKD (50.0% vs. 24.1%; p = 0.01). The cause of ESRD was more frequently a glomerulonephritis (48.7% vs. 37.2%; p = 0.04) or a nephroangiosclerosis (12.4% vs. 4.4%; p = 0.02) in the cases, while ADPKD (6.2% vs. 23.0%; p = 0.01) and uropathies (3.5% vs. 11.1%; p = 0.04) were more frequent in controls (Table 1). There was no difference in the immunosuppressive treatment and immunological complications before baseline (Table 2).

**TABLE 2:** Immunosuppressive treatments and immunological complications of cases and controls before baseline.

Variables	Patients	
	Cases n = 113	Controls n = 226
Induction therapy None Thymoglobulin Anti-IL2-R	2 (1.8) 69 (61.1) 42 (37.2)	5 (2.2) 134 (59.3) 87 (38.5)
Maintenance IS therapy Tacrolimus Ciclosporin Corticoids Mycophenolic acid Azathioprine mTOR inhibitors Belatacept	50 (44.2) 58 (51.3) 86 (76.1) 85 (75.2) 9 (8.0) 5 (4.4) 3 (2.7)	95 (42.0) 116 (51.3) 166 (73.5) 171 (75.7) 14 (6.2) 18 (8.0) 0 (0.0)
Dose/weight mycophenolate mofetil, mg/kg	17.3 (7.3)	16.1 (7.3)
Dose/weight mycophenolate sodium, mg/kg	10.6 (6.0)	13.2 (6.3)
Exposure time to CNI, months	40.8 (15.6–99.6)	44.6 (16.3–91.8)
Exposure time to TIT, months	18.0 (4.8–59.3)	14.6 (4.0–62.3)
Immunological complications Allograft nephropathy Active-AMR Chronic-AMR Acute-TCMR Acute rejection (type not specified) *De novo* DSA	10 (8.8) 2 (1.8) 1 (0.9) 2 (1.8) 6 (5.3) 2 (1.8)	22 (9.7) 5 (2.2) 1 (0.4) 8 (3.6) 9 (4.0) 7 (3.1)

Data are expressed as mean (standard deviation) or median (interquartile range) for continuous variables (depending on the normal distribution or not) and number (percentage) for categorial variables. Baseline correspond to date of cancer diagnosis for cases and for controls on the date of cancer of the matched case; IL2R: interleukin-2, receptor; mTOR: mechanistic target of rapamycin; CNI: calcineurin inhibitor; TIT: triple immunosuppressive therapy; AMR: antibody-mediated rejection; TCMR: T cell-mediated rejection; DSA: donor specific antibody. There was no significant difference between cases and controls (p-value >0.05 for all variables).

In the univariate analysis, male gender [OR 1.9; CI 95% (1.1–3.1); p = 0.01], ACKD [OR 3.4; CI 95% (2.0–5.7); p < 0.01], glomerulonephritis [OR 1.7; CI 95% (1.0–2.7); p = 0.01] and nephroangiosclerosis [OR 2.8; CI 95% (1.2–6.5); p = 0.02] as a cause of ESRD were associated with RCC, while ADPKD [OR 0.2; CI 95% (0.1–0.5); p < 0.01] and uropathies [OR 0.4; CI 95% (0.1–0.9); p = 0.04] were protective factors. Only male gender [OR 2.2; CI 95% (1.2–4.4); p = 0.02] and ACKD [OR 3.2; IC 95% (1.8–5.9); p < 0.001] remained associated with the risk of RCC in the multivariate analysis (see the Supplementary Table for comprehensive results).

**FIGURE 1:**
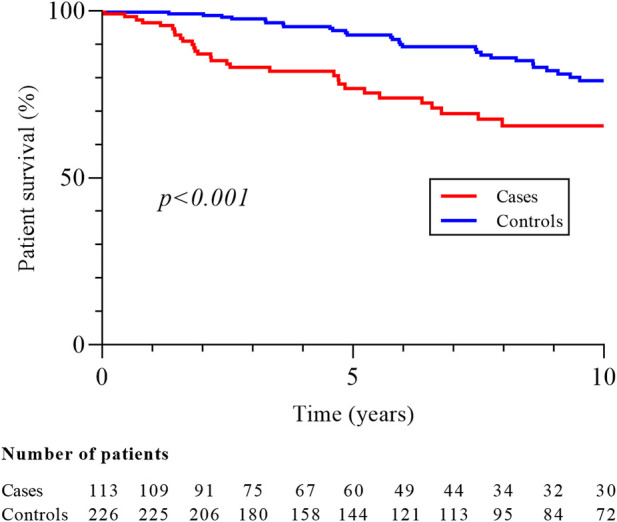
Patient survival in cases and controls groups. Patient survival was measured from baseline, corresponding date of cancer diagnosis for cases and for controls on the date of cancer of the matched case.

**FIGURE 2:**
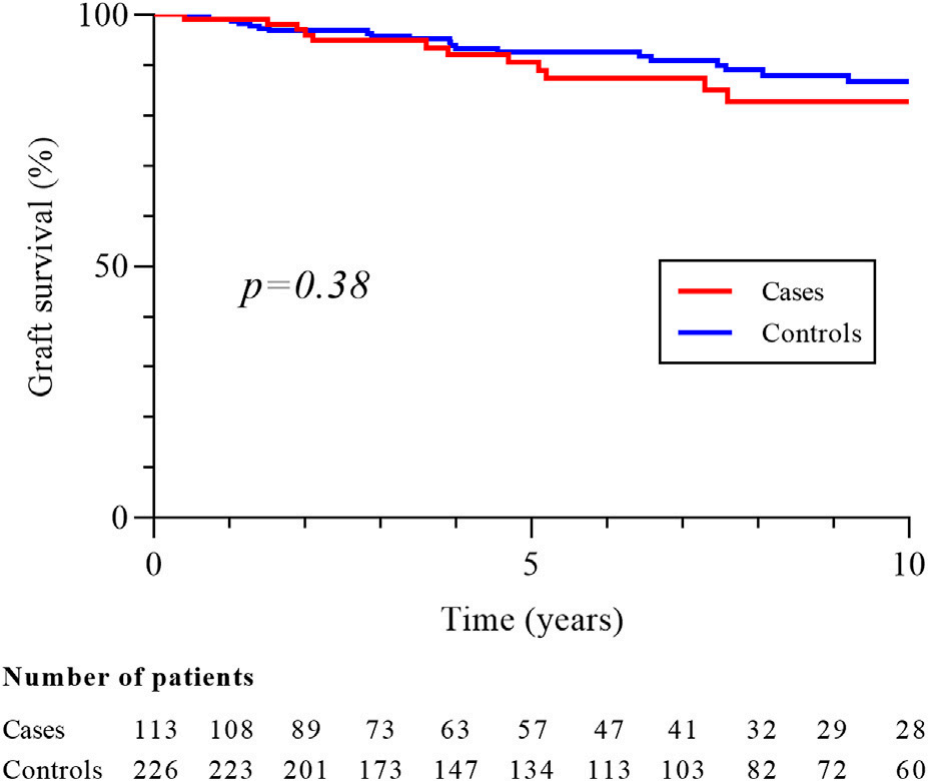
Graft survival in cases and controls groups. Graft survival is censored on patient death and measured from baseline, corresponding date of cancer diagnosis for cases and for controls on the date of cancer of the matched case.

With respective median follow-up times of 62.5 months (IQR 29.9–120.1) and 82.1 months (IQR 39.9–134.4), the mortality rate (33.6% vs. 18.6%, p = 0.02) and 10-year survival (65.6% vs. 79.1%, p < 0.001; Figure 1) were better in controls. Patients with metastatic disease at diagnosis had a median survival of 9.0 months (IQR 8.6–9.3). RCC was the second cause of death in cases (10/38, 26.3%) after cardiovascular disease (CVD; 12/38, 31.6%) while infections (13/42, 30.9%), malignancies (10/42, 23.8%) and CVD (9/42, 21.4%) were the most common causes of death in controls. There was no difference in the 10-year graft survival censored on patient death (82.7% in cases vs. 86.7% in controls, p = 0.38; Figure 2), and chronic allograft nephropathy was the most common cause of graft loss in the two groups (60.0% in cases and 65.2% in controls). Finally, the risks after baseline of allograft nephropathy (9.7% in cases vs 6.2% in controls), humoral rejection (7.1% in cases vs. 5.6% in controls), cellular rejection (4.4% in cases and 1.3% in controls) and appearance of DSA (14.2% in cases and 13.5% in controls) were similar between the two groups.

### RCC Characteristics

**FIGURE 3:**
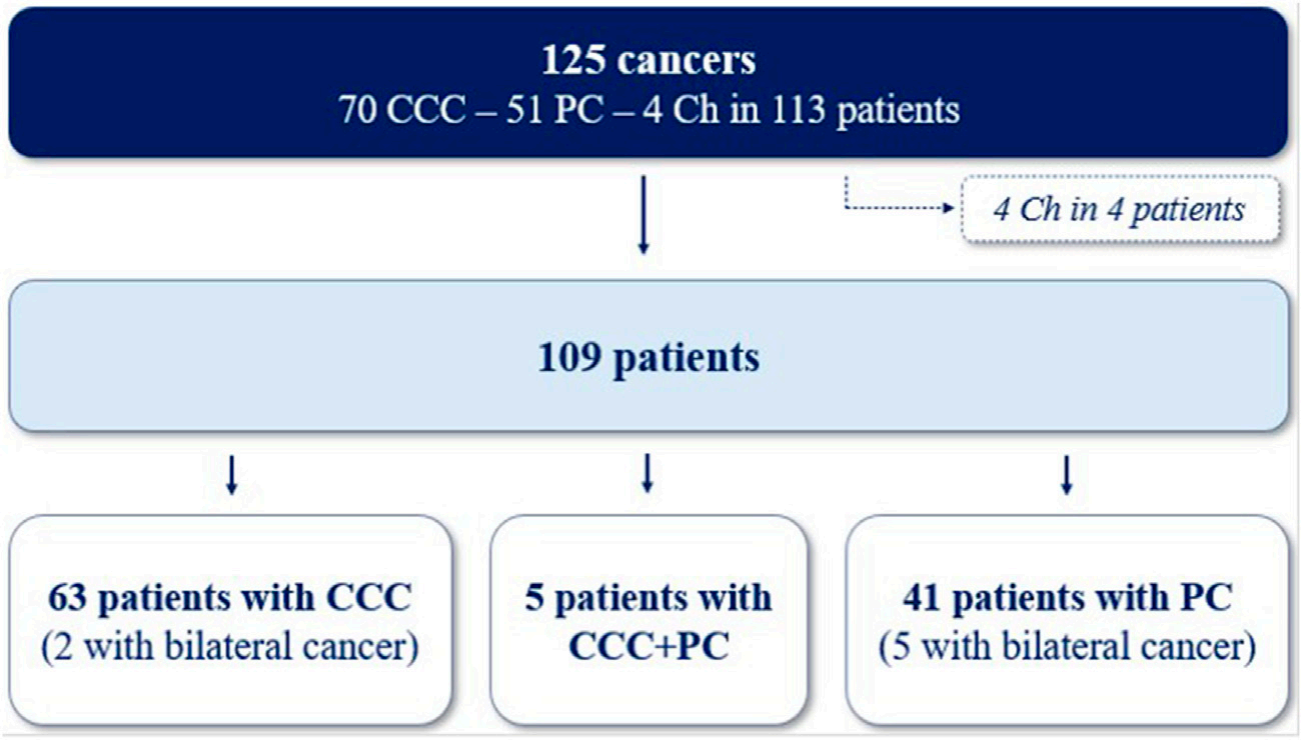
Repartition of 125 cancers in 113 patients. CCC, clear-cell carcinomas; PC, papillary carcinomas; Ch, chromophobes cancers.

**FIGURE 4:**
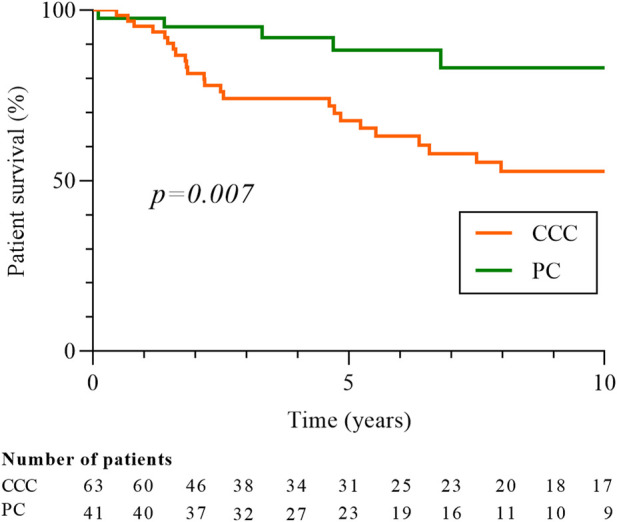
Patients’ survival according to histologic type of RCC, clear-cell or papillary carcinomas. Patient survival is measured from date of cancer diagnosis. Five patients with CCC and PC were excluded from the analysis. CCC: clear-cells carcinomas; PC: papillary carcinomas.

One hundred and twenty-five cancers were diagnosed in the 113 cases (Figure 3). Clear cell carcinoma (CCC) was the most common type of cancer (56.0%), followed by papillary (PC; 40.8%) and chromophobe subtypes (Ch; 3.2%). Due to the low number of Ch cancers, only CCC and PC were included in the following analysis. The median time between cancer diagnosis and transplantation was 38.4 months (17.3–84.3) for CCC and 42.1 months (15.2–102.2) for PC. There was no difference in the clinical characteristics according to the histological subtypes, except for ciclosporin use which was more frequent in patients with CCC (61.9% vs 36.6%; p = 0.01). Most cancers were low stage (stage T1-T2, 94.2%) and low grade (grade 1 or 2, 63.0%) at diagnosis in both types of cancer and there was no difference between CCC and PC for tumor size, stage, grade and focality (Table 3). Despite this, 10-year survival was worse in patients with CCC (52.8% vs 83.1%, p = 0.007; Figure 4), and the rate of cancer-related deaths had a clear tendency to be higher (33.3% vs. 0.0%, p = 0.07).

**TABLE 3:** Histological characteristics of clear-cells and papillary carcinomas.

Variables	Cancers	
	Clear cells n = 70	Papillary n = 51
Tumor size, mm	23.5 (15.5–37.5)	22.0 (15.0–33.0)
TNM stage T1-T2 T3-T4 N+ M+	64 (91.4) 6 (8.6) 1 (1.4) 2 (2.9)	50 (98.0) 1 (2.0) 0 (0.0) 0 (0.0)
Grade^a^ G1-G2 G3-G4	38 (62.3) 23 (37.7)	25 (64.1) 14 (35.9)
Multifocal	12 (17.1)	16 (31.4)

Data are expressed as median (interquartile range) for continuous variables and number (percentage) for categorial variables. Ns: non-significant.

^a^ 21 patients with unknown histological grade (9 in Clear cells group and 12 in Papillary group) were excluded from the analysis. There was no significant difference between two groups (p-value >0.05 for all variables).

### Treatment and Relapse

All cancers were treated by nephrectomy. A contralateral preventive nephrectomy was performed in 19 patients resulting in the diagnosis of 6 cancers. The immunosuppressive treatment was mostly unmodified after the surgery (68.0%) and 24 patients (19.2%) were switched to an mTOR inhibitor. Only one patient (with metastatic disease at diagnosis) received anti-angiogenic therapy. Ten patients (9.0%) relapsed after surgery, among whom 9 had metastases (Table 4). The median delay of relapse was 9.5 months (4.5–47.7). Patients were mostly men (90.0%), aged over 60 at diagnosis (80.0%) and all these patients had CCC (relapse rate: 16.4% for CCC and 0.0% for PC, p = 0.002). Changes in immunosuppressive treatments after relapse were not different from those across the cohort. This recurrence was treated by antiangiogenic therapy in 4 patients (2 with Sunitinib and 2 unspecified, 40.0%), and by an mTOR inhibitor in 3 (30.0%). Eight patients (80.0%) died after this relapse, mainly due to the progression of RCC (75.0%). The median delay between death and relapse was 24 months (19.7–44.9) and 21.6 months (17.0–27.0) for patients who died from RCC.

**TABLE 4:** Characteristics of patients with relapse.

No	Sex	Age^a^	IT^a^	Histology	Stage	Grade	Relapse (delay)	Treatment	Death (cause)
1	M	63	Cs, MMF, CT	CCC	T1bN0	G2	M+, lung, bones (47 months)	Addition of imTOR	Yes (INF)
2	M	62	Tac, MMF, CT	CCC	T1aN0	G2	M+, pancreas (85 months)	Palliative care	No
3	M	61	Cs, MMF	CCC	T1aN0	G1	M+, lung, liver, bones (6 months)	Stop of Cs for imTOR	Yes (RCC)
4	M	68	Tac, MMF	CCC	T1aN0	G2	M+, lung, bones, brain (12 months)	Stop of MMF	Yes (RCC)
5	M	62	Tac, MS, CT	CCC	T3N0	G4	M+, lung (5 months)	Addition of imTOR	Yes (RCC)
6	M	65	Cs, CT	CCC	T3N0	G3	Local (7 months)	Surgical	Yes (CVD)
7	F	63	Tac, CT	CCC	T1aN0	G3	M+, lung, bones (22 months)	Sunitinib	Yes (RCC)
8	M	44	Cs, MMF, CT	CCC	T3N0	G3	M+, bones (3 months)	Sunitinib	Yes (RCC)
9	M	65	Tac, MMF, CT	CCC	T3N0	G3	M+, pleura (2 months)	AAG	Yes (RCC)
10	M	51	Tac, MMF	CCC	unknown	unknown	M+, bones (50 months)	AAG	No

IT: immunosuppressive treatments; M: male; F: female; Cs: ciclosporin; MMF: mycophenolate mofétil; CT: corticoids; Tac: tacrolimus; CCC: clear-cell carcinoma; M+: metastasis; imTOR: inhibitor of mechanistic target of rapamycin; AAG: anti-angiogenic therapy; INF: infection; RCC: renal cell carcinoma; CVD: cardiovascular disease.

^a^ Data at baseline.

### Impact of Screening Strategy

**FIGURE 5:**
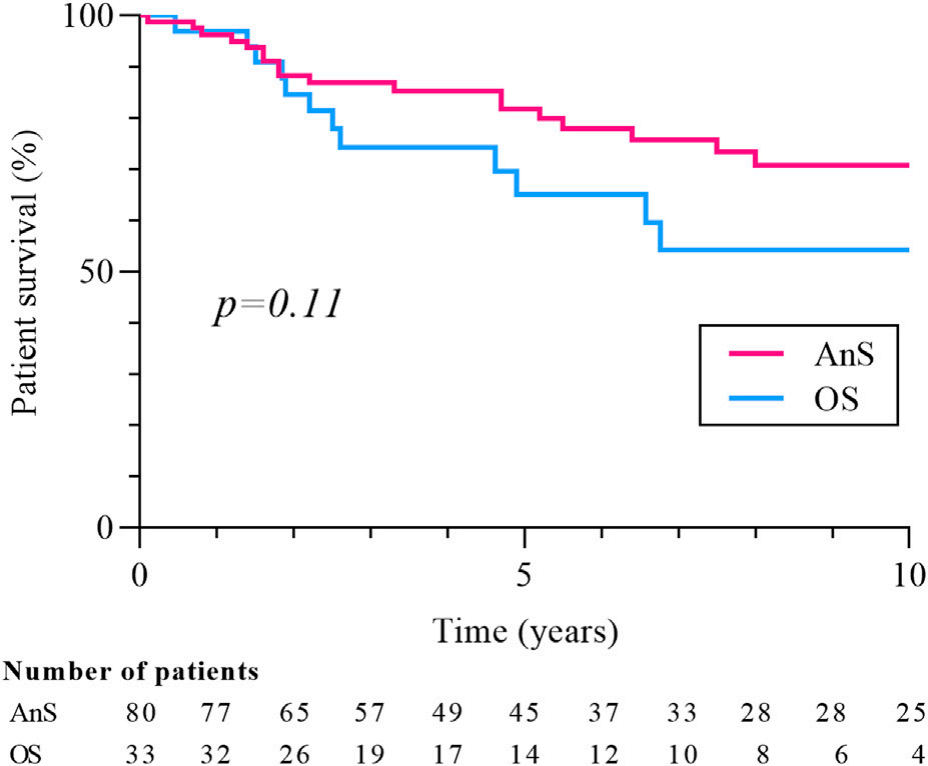
Patients’ survival according to screening strategy. Patient survival is measured from date of cancer diagnosis. Ans, annual screening, OS, other strategy.

Eighty-seven cancers were diagnosed in the 80 patients from the AnS-group and 38 in the 33 patients of the OS-group. The two patients with a history of pre-transplant RCC were in the AnS group, and their screening strategy did not differ from that of the rest of the group. The prevalence of RCC was 2.7% in the AnS-cohort and 0.8% in the OS-cohort (p < 0.0001). The median time between cancer diagnosis and transplantation was 40.5 months (15.3–90.8) in the AnS-group and 46.4 months (21.1–101.5) in the OS-group (p = 0.34). Patients in the AnS-group (compared to-OS group) were younger at baseline (54.7 ± 10.5 vs. 59.5 ± 10.4 years, p = 0.03), more frequently dialyzed before transplantation (97.5% vs. 84.8%, p = 0.01), had more ciclosporin (60.0% vs. 30.3%, p = 0.004), corticoids (81.2% vs. 63.6, p = 0.04), less tacrolimus (33.7% vs. 69.7%, p < 0.001), and a lower dose of mycophenolate mofetil [dose/weight ratio: 14.4 (11.8–19.9) vs. 17.6 (14.3–25.3) mg/kg, p = 0.02]. Similar to the entire cohort, CCC remained the main histological subtype in each group (57.5% in AnS and 52.6% in OS, p = 0.61). Stage and grade were similar between the two cohorts, but cancers were significantly smaller in the AnS-group [median tumor size: 20.0 (15.5–32.5) vs. 30.0 (20.0–40.0) mm, p = 0.01]. Both patients with metastasis at diagnosis were in the AnS group, at the center alternating CT and ultrasound screening. Their last negative screening test was an ultrasound, performed respectively 3 and 12 months prior to the diagnosis of metastatic RCC. Relapses were less frequent in the AnS group (5.0% vs. 18.2%, p = 0.02) and the time between cancer diagnosis and relapse tended to be longer [29.5 (7.9–75.4) vs. 6.0 (2.7–29.0) months, p = 0.26). Finally, mortality rate (31.2% vs 39.4%, p = 0.40) and 10-year survival (70.3% vs 54.2%, p = 0.11, Figure 5] did not differ between the two groups, but the rate of cancer-related deaths was significantly lower in the AnS group (16.0% vs. 46.1%, p = 0.04).

## Discussion

This retrospective study reports the characteristics and outcome of 113 patients who presented RCC out of a population of just over 7,000 KTRs in four University Hospitals. One hundred and twenty-five cancers were diagnosed in this population during a 27-year period, mostly clear cell carcinomas at low grade and early stage. We identified two risk factors for the occurrence of native kidney cancer in KTRs: male gender and ACKD. RCC was associated with a reduction in patient survival but did not influence graft survival or immunological complications. Mortality was particularly high in patients with disseminated disease, whether at diagnosis or at relapse. Clear cell carcinoma was associated with a poorer prognosis, reduced survival and increased risk of recurrence compared to papillary carcinoma. We focused on the impact of screening and showed that patients in centers with annual screening had smaller cancers, a lower relapse rate, and a lower rate of cancer-related deaths.

Several studies have analyzed the risk of occurrence of RCC during KT, but most of them included only a small number of patients [16–27]. Moreover, these studies did not compare the characteristics of these patients to a control population, making it impossible to study the risk factors of RCC in KT. This work is, to our knowledge, the first study comparing patients with RCC to a control population of KTRs without RCC and is also one of the cohorts with the largest number of patients.

The recognized risk factors for RCC in the general population are age over 60, male gender, smoking, high blood pressure, obesity, and certain genetic disorders [5–12]. In the present study, only male gender and the presence of ACKD remain associated with an increase-risk of RCC in the multivariate analysis. Several potential RCC risk factors, including smoking quantification and family history, could not be assessed due to missing data inherent to the study design. Several studies have suggested an association between ACKD and RCC, due to the high prevalence of this anomaly in ESRD patients with RCC (70%–90%) [13, 17, 32]. ACKD and its potential link to RCC are not fully understood. Nephron reduction associated with renal failure could lead to the expression of growth factors (such as *Epidermal Growth Factor* and *Hepatocyte Growth Factor*) and proto-oncogenes (such as c-jun), which can promote the hypertrophy and hyperplasia of tubular cells (contributing to the appearance of cysts), but also the development of RCC [13]. No difference in immunosuppression or immunological complications before baseline was observed between cases and controls. However, our analysis was mainly qualitative, as accurately quantifying immunosuppression remains challenging. Use of emerging biomarkers of immunosuppression levels, like Torque Teno Virus viremia, may offer a better assessment of the link between immunosuppression and RCC development [39, 40].

In line with previous studies RCC were mostly at low stage and low grade, and had a lower rate of relapse than in the general population (30.0%–40.0%) [35]. Despite these good outcomes we also showed that KTRs with RCC had a lower survival rate than controls. However, the higher frequency of males in RCC patients could partly explain this difference. Clear cell carcinomas were particularly associated with a poor prognosis, reflected by more recurrence and a higher mortality than papillary cancers. This poorer outcomes were already reported in the general population and could be related with worse stage and histological grade at diagnosis [41, 42], however we did not highlight any difference on these criteria in our analysis.

All cancers were managed by nephrectomy and no patient received alternative treatment such as radiofrequency ablation, cryotherapy, or active surveillance. Biopsy followed by active surveillance remains a possible option in transplant recipients, especially in frail patients or those with high surgical risk [29]. The management of immunosuppressive therapy after the diagnosis of cancer is an important concern for Transplant-nephrologists. We showed that the overall immunosuppression was modified in one-third of the patients, mainly for the use of a mTOR inhibitor. mTOR inhibitors indeed have immunosuppressive and anti-cancer effects, making them interesting in the treatment of post-transplant cancers [2]. However, the use of mTOR inhibitors in this indication has only been validated in the context of non-melanoma skin cancers and Kaposi sarcoma, moreover these treatments have lower immunosuppressive effects than CNI and can lead to the increase in proteinuria. Furthermore, mTOR inhibitors are used in the general population in the treatment of metastatic RCC, but they are not used as adjuvant treatment of localized cancers. Based on these elements and results of the present study, our opinion is that the use of an mTOR inhibitor in KTRs has a strong rational in the context of metastatic RCC and can be discussed in localized clear cell carcinomas (which have in the present study an increased risk of recurrence and mortality). Conversely, the best prognosis of papillary subtype that we report does not tend to offer these therapies in patients with localized papillary cancer.

The benefit of CRN screening in KTRs remains subject to debate. Although our study was not designed to evaluate the benefit of a screening procedure, we studied the association between the annual screening with abdominal imaging and the outcomes of KTRs with RCC. Our data suggest that annual screening is associated with earlier cancer diagnosis, leading to fewer relapses and fewer cancer-related deaths. However, we were not able to demonstrate a statistical link between annual screening and a better survival. From our point of view, there was a clear trend towards better survival in the AnS-group, and this result could be explained by a lack of statistical power linked to the low numbers in the OS-group. Despite these elements, systematic screening for RCC appears to have several limitations. Indeed, in our study, two patients undergoing annual screening presented with metastasis at diagnosis, and none in the OS-groups. Interestingly, both patients had a negative ultrasound as their last screening test, raising concerns about the sensitivity of this modality for RCC screening in transplant recipients. In addition, 19 preventive nephrectomies (therefore without visible anomaly on imaging) were performed in our study and almost a third of these allowed the incidental diagnosis of another cancer, which may suggest a lack of sensitivity of imaging tests. More of that, Tillou *et al,* presented a series of 21 RCCs from 31 patients in whom an imaging examination (ultrasound or scanner) had detected a suspicious lesion [23]. Here, almost a third of the operated patients (10/31, 32.3%) ultimately did not have cancer, also suggesting a risk of low specificity of imaging tests. Thus, our encouraging results regarding annual screening need to be confirmed in future work and on a larger cohort. Given the existing doubt about the benefit of RCC screening in KTRs, it seems appropriate to target this screening in patients at risk (notably in males and patients with ACKD).

This study has limitations and biases. Due to the retrospective design of the work, there are probably missing or incomplete data, particularly for older ones. We analyzed RCCs occurring after transplantation; however, despite thorough screening before transplantation, some tumors—especially those diagnosed soon after transplantation—may have preexisted. In addition, the high prevalence of native kidney cancer in one of the four centers may cause a “center-effect.” More, we had a long study period, which could have led to heterogeneity in the definitions, classifications and patient care. Finally, we presented the outcome of patients with RCC as well as the centers’ screening policy but did not collect the type of follow-up after the cancer diagnosis. A difference in monitoring that could interfere with the outcome of patients.

## Conclusion

This work made it possible to better clarify the characteristics and outcome of kidney transplant recipients who developed native kidney cancer. Most cancers were localized at the time of diagnosis and recurrence after nephrectomy was rare. However, the prognosis of patients with disseminated disease was poor and survival of cases was lower than that of controls. Clear cell carcinomas had a particularly poor prognosis than papillary carcinomas and a modification of immunosuppressive treatment should be discussed. Finally, patients benefiting from annual screening tended to have cancers with better characteristics. The benefit of screening requires studies with larger numbers. As male sex and acquired cystic kidney disease are associated with native kidney cancer, these populations could constitute an interesting target for the screening.

## Data Availability

The data analyzed in this study is subject to the following licenses/restrictions: All data are available on request from the corresponding author. Requests to access these datasets should be directed to pommerolle.pierre@chu-amiens.fr.

## Abbreviations

ACKD, acquired cystic kidney disease; Ans, annual screening group; ADPKD, autosomal dominant polycystic kidney disease; CVD, cardiovascular disease; Ch, chromophobe; CCC, clear cell carcinoma; CKD, chronic kidney disease; DSA, donor specific antibody; ESRD, end-stage renal disease; eGFR, estimated glomerular filtration rate; KT, kidney transplantation; KTRs, kidney transplant recipients; Os, other strategy group; PC, papillary carcinoma; RCC, renal cell carcinoma.
