# Supplementary material for: Renal Cell Carcinoma in Native Kidney After Kidney Transplantation: A Multicenter Case Control Study With a Focus on Screening Strategy
Source: Transpl Int. 2025 Jun 16;38:14487. doi: 10.3389/ti.2025.14487 (PMC12206669; doi:10.3389/ti.2025.14487)
Supplement: Supplementary file 1 [file Table1.docx]

Table. Univariate and multivariate logistic regression analysis of factors associated with renal cell carcinoma.

|  | Univariate | | | Multivariate* | | |
| --- | --- | --- | --- | --- | --- | --- |
|  | **OR** | **IC 95%** | **p** | **OR** | **IC 95%** | **p** |
| Male | 1.9 | [1.1 - 3.1] | 0,01 | 2.2 | [1.2 - 4.4] | **0.02** |
| Acquired cystic kidney disease | 3.4 | [2.0 - 5.7] | 0.001 | 3.2 | [1.8 – 5.9] | **0.0001** |
| Prior Transplant | 1.7 | [0.8 - 3.4] | 0.14 |  |  |  |
| Time to dialysis >3 years | 1.6 | [0.9 - 2.6] | 0.06 |  |  |  |
| Glomerulonephritis | 1.7 | [1.0 - 2.7] | 0.01 |  |  |  |
| Nephroangiosclerosis | 2.8 | [1.2 - 6.5] | 0.02 |  |  |  |
| Urologic malformation | 0.4 | [0.1 - 0.9] | 0.04 |  |  |  |
| Autosomal dominant polycystic kidney disease | 0.2 | [0.1 - 0.5] | 0.001 |  |  |  |
| Smoke | 1.0 | [0.6 - 1.6] | 0.93 |  |  |  |
| Prior cancer history | 0.6 | [0.3 - 1.4] | 0.24 |  |  |  |
| Thymoglobulin | 1.1 | [0.7 - 1.7] | 0.75 |  |  |  |
| Calcineurin inhibitor | 1.1 | [0.4 - 2.7] | 0.87 |  |  |  |
| mTOR inhibitor | 0.5 | [0.2 - 1.5] | 0.23 |  |  |  |

* Including variables with p<0.1 in univariate analysis.
